# Anti-inflammatory activity of *Ternstroemia gymnanthera* stem bark extracts in bacterial lipopolysaccharide-stimulated RAW264.7 murine macrophage cells

**DOI:** 10.1080/13880209.2017.1278778

**Published:** 2017-01-31

**Authors:** Thamizhiniyan Venkatesan, Eun-Jin Park, Young-Woong Choi, Jennifer Lee, Young-Kyoon Kim

**Affiliations:** Department of Forest products and Biotechnology, College of Forest Science, Kookmin University, Seoul, South Korea

**Keywords:** Ornamental plant, traditional medicine, inflammation, natural antioxidants

## Abstract

**Context:***Ternstroemia gymnanthera* Sprague (*Theaceae*) possesses various known pharmacological properties. However, its anti-inflammatory activity has not been reported.

**Objective:** The anti-inflammatory activity of *Ternstroemia gymnanthera* stem bark aqueous extract (TGSBE) was evaluated using LPS-stimulated RAW264.7 macrophages.

**Materials and methods:** Cytotoxicity was assessed by MTT assay after 24 h with TGSBE (25–200 μg/mL). Further testing used TGSBE at 100 and 200 μg/mL. Griess and ELISA methods after 24 h with TGSBE determined NO and cytokine levels, respectively; then, mRNA levels (iNOS & cytokines) were analyzed by Quantitative-PCR after 12 h. NF-κB and MAPK were assessed by immunoblotting after TGSBE treatment for 12 h, followed by LPS for 30 min. Immunofluorescence assay was also performed for NF-κB. ROS and MMP, after 12 h with TGSBE, were determined by flow cytometry. The antioxidant potential of TGSBE was analyzed by ABTS assay. The Folin–Ciocalteu method determined the total phenolic content of TGSBE. LPS concentration was 0.5 μg/mL.

**Results:** TGSBE at 200 μg/mL showed about 96.2% viability while suppressing the production of NO (88.99%), TNFα (24.38%), IL-6 (61.70%) and IL-1β (55.12%) and gene expression by 67.88, 45.24, 65.84, and 70.48%, respectively. TGSBE decreased ROS (79.26%) and improved MMP (48.01%); it inhibited translocation of NF-κB and MAPK activation. Radical scavenging activity was 50% at 402.17 μg/mL (ascorbic acid standard: 88.8 μg/mL). Total phenolic content was 240.9 mg GAE/g.

**Discussion and conclusion:** TGSBE suppresses the inflammatory response by inhibiting the NF-κB and MAPK cascades exhibiting therapeutic potential to treat inflammatory diseases associated with increased activation of macrophages.

## Introduction

Inflammation is a natural host defense process commonly initiated against invading foreign pathogens and in response to various systemic injuries (Bistrian 2007; Mogensen 2009). During this process, the most important acute inflammatory cells and macrophages are activated. These cells, then, produce various inflammatory mediators, particularly reactive oxygen species (ROS), nitric oxide (NO), prostaglandin E_2_ (PGE_2_), tumor necrosis factor-α (TNFα), interleukin-6 (IL-6), and interleukin-1β (IL-1β). These mediators amplify the inflammatory response either to successively eliminate invading foreign pathogens or remodel damaged tissue structures. However, prolonged increased production of these inflammatory mediators has damaging effects to multiple organs (Laskin & Pendino 1995). Many investigations have clearly demonstrated the contribution of chronic activation of macrophages in the pathogenesis of various inflammatory diseases such as cardiovascular disease, osteoporosis, autoimmune diseases, sepsis-related multi-organ failure, neurodegenerative disorders, and tumorigenesis (Jou et al. 2013). Thus, the inhibition of macrophage activation is a potential method for ameliorating the severity of inflammatory diseases.

*Ternstroemia gymnanthera* Sprague (Theaceae) is an ornamental plant that commonly grown in gardens in many countries such as Japan, China, Korea, Taiwan and India. The leaves of the plant have long been used to treat malaria in Taiwan. Additionally, the bark and root of this plant are reported to possess astringent and anticancer properties (Ikuta et al. 2003). Since the anti-inflammatory activity of *Ternstroemia gymnanthera* has not yet been reported, we investigated the effects of *Ternstroemia gymnanthera* stem bark aqueous extract (TGSBE) on bacterial lipopolysaccharide (LPS) stimulated RAW264.7 murine macrophage cells. LPS activates a set of intracellular signaling pathways including NF-κB and MAPKs pathways in macrophages that regulate the expression of genes encoding various inflammatory mediators. LPS additionally increases the production of reactive oxygen species (ROS) and subsequently amplifies the MAPKs and NF-κB activation, as well as damages the mitochondrial membrane integrity (Dobrovolskaia & Vogel 2002; Haddad & Land 2002; Kim et al. 2010). Hence, the modulatory effects of TGSBE on the LPS-induced production of inflammatory mediators, ROS, MMP and related intracellular signaling pathways were investigated in this study.

## Materials and methods

### Materials

The stem bark of *Ternstroemia gymnanthera* was collected during the month of March, 2016 from the Wando Island in the Republic of Korea. The plant was identified and authenticated by the corresponding author Prof. Young-Kyoon Kim. A voucher specimen (TGSB-016) was deposited in the herbarium of the College of Forest Science, Kookmin University, Korea. The murine macrophage RAW264.7 cell line was obtained from the Korea Cell Line Bank, Seoul, Korea. LPS (*E. coli*, Serotype 0111:B4), MTT, DCFH-DA, JC-1 and ABTS were purchased from Sigma-Aldrich (St. Louis, MO). ELISA kits for cytokine assay were from R&D Systems (Minneapolis, MN). The primary antibodies for iNOS, NF-κB p65, Lamin-B and GAPDH were from Santa Cruz Biotechnology, Inc. (Dallas, TX). Anti-p38, anti-phospho-p38, anti-JNK, anti-phospho-JNK, anti-ERK, anti-phospho-ERK, anti-Akt, anti-phospho-Akt, were purchased from Cell Signaling Technology (Beverly, MA). iScript^TM^ cDNA Synthesis kit and iQ^TM^ SYBR® Green Super mix were from Bio-Rad Laboratories, Inc. (Bio-Rad, PA). Primers for PCR were purchased from Bioneer Corporation (Korea). All other chemicals and reagents were of the highest grade available on the market.

### Cell culture

RAW264.7 cells were grown in DMEM supplemented with 10% heat-inactivated fetal bovine serum (FBS), 100 U/mL penicillin, 100 μg/mL streptomycin sulfate and 0.25 μg/mL amphotericin B. Cultures were maintained at 37 °C in a humidified atmosphere of 5% CO_2._

### Extraction and fractionation

The stem bark was washed and shade dried at room temperature for one week. Then, the stem bark (500 g) was ground with an electronic laboratory blender and extracted with distilled water (3 L) using a Soxhlet apparatus at 100 °C. The extracts were concentrated under reduced pressure using a rotary evaporator and lyophilized (yield, approximately 16%). The stock solution of *Ternstroemia gymnanthera* stem bark extract (TGSBE) was prepared using DMSO at a concentration of 50 mg/mL, filter sterilized using 0.2 μm, DMSO-Safe Acrodisc® Syringe Filter (Pall Corporation, NY, USA), twice, and stored at −20 °C for further use.

### Cell viability assay

To determine the cytotoxic effect of TGSBE on RAW264.7 macrophage cells, an MTT colorimetric assay was performed as described previously (Wu et al. 2015). Briefly, cells were seeded into 96-well plates at a density of 1 × 10^5^ cells/well and were treated with various concentrations (25, 50, 100 and 200 μg/mL) of TGSBE for 24 h. Afterwards, media was discarded and, 100 μL of fresh media containing MTT (0.5 mg/mL) was added to the cells. Incubation was continued for 3 h. After removing the medium, the formazan crystals were dissolved in DMSO, and the absorbance was measured at 570 nm on a micro-plate reader.

### Nitric oxide assay

Cells (1 × 10^5^ cells/well) were seeded into 96-well plates and allowed to grow for 24 h. Cells were pre-incubated in the presence or absence of TGSBE for 1 h, then cells were exposed to 0.5 μg/mL of LPS for 24 h. After incubation, the cell-free supernatant was mixed with an equal volume of Griess reagent (0.1% *N*-(1-naphthyl) ethylenediamine in distilled water and 1% sulfanilamide in 5% phosphoric acid, 1:1 ratio). After a 10 min incubation period, absorbance was measured at 540 nm on a micro-plate reader.

### Determination of TNF-α, IL-6, and IL-1β production

Cells were pre-incubated with TGSBE for 1 h, and then exposed, or not, to 0.5 μg/mL of LPS for 24 h. After incubation, the cell-free supernatant was collected to determine the levels of cytokines, TNF-α, IL-6, and IL-1β using commercial ELISA kits, according to the manufacturer’s protocol.

### Determination of intracellular reactive oxygen species

Cells were seeded at 1 × 10^6^ cells/mL into a 6-well plate and incubated for 24 h. Following a 1 h pretreatment with TGSBE (0, 100 and 200 μg/mL), and then with or without LPS for 12 h, the cells were incubated with the oxidation-sensitive fluorescent probe, DCFH-DA (10 μM) for 30 min. After the removal of excessive DCFH-DA, cells were washed with PBS and analyzed using a flow cytometer.

### Detecting mitochondrial membrane potential (MMP)

Cells were seeded at 1 × 10^6^ cells/mL into a 6-well plate and incubated overnight. After subjecting the cells to the appropriate treatment, cells were incubated with 1 μg/mL of JC-1 in fresh medium for 15 min. Subsequently, cells were washed with phenol-red free media twice and MMP was analyzed by flow cytometry.

### ABTS radical scavenging assay

ABTS radical scavenging activity of TGSBE was determined according to the method of Re et al. (1999) with some modifications. Briefly, ABTS radical was produced by reacting ABTS (7 mM in water) with potassium persulfate (2.45 mM) in the dark at room temperature for 12 h. Then, the solution was diluted with ethanol to give an absorbance of 0.7 at 734 nm. To 1.0 mL of ABTS radical solution, 20 μL of different concentrations (0, 31.25, 62.50, 125, 250 and 500 μg/mL) of TGSBE or ascorbic acid in distilled water, was added and after 1 min, the decrease in absorbance was measured at 734 nm. The percentage radical scavenging activity was calculated as: % ABTS radical scavenging = [(Control – Test/Control) × 100].

### Total phenolic content assay

The total phenolic content of TGSBE was determined by the method described previously with some modifications (Sone et al. 2011). Briefly, 100 μg of TGSBE in 1 mL of distilled water was mixed with 0.5 mL of Folin–Ciocalteu reagent (1:1 ratio, diluted in distilled water) and allowed to stand for 3 min. Then, 2.5 mL of 20% (w/v) sodium carbonate was added and the mixtures were incubated at dark for 1 h. The absorbance was measured at 765 nm. The total phenolic content was calculated from the calibration curve prepared with gallic acid. The total phenolic content of TGSBE was 240.9 mg of gallic acid equivalent (GAE) per gram dry weight.

### Immunoblotting assay

Total protein was extracted from RAW264.7 macrophage cells by RIPA buffer supplemented with protease and phosphatase inhibitors (Roche). Cytosolic and nuclear fractionation was performed as described previously. Briefly, the cell pellets were mixed with 400 μL hypotonic buffer (10 mmol/L HEPES at pH 7.9, 10 mmol/L KCl, 1.5 mmol/L MgCl_2_, 0.1 mmol/L EDTA, 0.5 mmol/L DTT, and protease and phosphatase inhibitors). Solutions were then placed on ice for 15 min, supplemented with NP-40 to a final concentration of 0.1%, agitated for 15 s, and centrifuged for 1 min at 4 °C and 12000 rpm. The supernatant was taken as the cytosolic protein extract and the salt concentration was adjusted to 100 mmol/L. The resulting pellets were washed twice with hypotonic buffer without detergent and mixed with 25 μL nuclear lysis buffer (HEPES 20 mmol/L at pH 7.9, NaCl 420 mmol/L, MgCl_2_ 1.5 mmol/L, EDTA 1.0 mmol/L, DTT 1.0 mmol/L, and protease and phosphatase inhibitors), placed on ice for 15 min, agitated for 15 s, and centrifuged for 15 min at 4 °C and 12000 rpm. The supernatant was taken as the nucleoprotein extract. To reduce the salt concentration, the nuclear extract was mixed with 50 μL of buffer (HEPES 20 mmol/L at pH 7.9, protease and phosphatase inhibitors). The protein concentration of the extracts was determined using BCA protein assay kit (Sigma). Volumes of the extracts were adjusted to contain equal protein concentrations with the same lysis buffer. Equal amounts of protein from each sample was taken for SDS-PAGE and transferred to a nitrocellulose membrane. The membrane was blocked with 5% (w/v) nonfat milk or 5% (w/v) BSA for 1 h, and then incubated with primary antibodies (iNOS, NF-κB p65, Lamin-B, GAPDH, p38, phospho-p38, JNK, phospho-JNK, ERK, phospho-ERK, Akt, or phospho-Akt) at 4 °C overnight. The membrane was incubated in horseradish peroxidise conjugated secondary antibodies at room temperature for 1 h, and then developed using the ECL method. The results were recorded with a LI-COR image processing system, and analyzed with gel image systems software.

### Fluorescent microscopy

Cells (1 × 10^6^cells/mL) were seeded on poly-l-lysine-coated glass coverslips placed in 6-well plates and allowed to grow for 24 h. Cells were pre-incubated to TGSBE for 12 h, and then stimulated with LPS (0.5 μg/mL) for 30 min. Cells were washed with ice cold-PBS (pH, 7.2) and fixed in 4% (w/v) paraformaldehyde in PBS for 20 min. Cells were permeabilized with 0.2% (v/v) Triton X-100 for 10 min, and blocked 3% (w/v) BSA for 30 min at room temperature. Next, cells were incubated with primary antibody (p65) overnight at 4 °C and washed with 0.01% Triton-PBS. Cells were incubated with FITC-conjugated secondary antibody for 30 min in the dark. Cells were washed three times with PBS-T (0.05% Tween 20), incubated with hoechst33342 (2 μg/mL) for 10 min, and then washed three times with PBS-T. Cells were examined and images were captured under fluorescent microscope.

### Quantitative real-time RT-PCR analysis

Cells were pretreated with TGSBE for 1 h and then stimulated with 0.5 μg/mL of LPS for 12 h. Total RNA was extracted with Trizol reagent according to the manufacturer’s instructions. For each sample, 1 μg of RNA was reverse transcribed to synthesize cDNA using an iScript^TM^ cDNA Synthesis kit (Bio-rad). To determine the mRNA levels of iNOS, TNFα, IL-6, and IL-1β, PCR amplification of cDNA was performed using specific primers in the customer iQ^TM^ SYBR^®^ Green Super mix (Bio-rad) with the following conditions: 95 °C for 3 min, followed by 40 cycles of 95 °C for 10 s, 60 °C for 30 s, 72 °C for 30 s. A melt curve analysis of target mRNAs was also performed to recognize the nonspecific signals. The expression levels of the gene of interest was calculated from triplicate measurement and normalized with the mean C_t_ of a control gene, β-actin.

The PCR primers used in this study were: iNOS, CAT GCT ACT GGA GGT GGG TG (forward), CAT TGA TCT CCG TGA CAG CC (reverse); TNFα, AGC ACA GAA AGC ATG ATC CG (forward), CTG ATG AGA GGG AGG CCA TT (reverse); IL-6, GAG GAT ACC ACT CCC AAC AGA CC (forward), AAG TGC ATC ATC GTT GTT CAT ACA (reverse); IL-1β, TGC AGA GTT CCC CAA CTG GTA CAT C (forward), GTG CTG CCT AAT GTC CCC TTG AAT C (reverse); and β-actin, ATC ACT ATT GGC AAC GAG CG (forward), TCA GCA ATG CCT GGG TAC AT (reverse).

### Statistical analysis

Data were analyzed using the prism software (GraphPad, San Diego, CA). Data were expressed as the mean ± SD of three independent experiments. A *p* value of less than 0.05 was considered statistically significant.

## Results

### Effects of TGSBE on the viability of RAW264.7 cells

No cytotoxicity was observed when cells were exposed to TGSBE (25–200 μg/mL) for 24 h (data not shown). Hence, we excluded the possiblity that anti-inflammatory effects were caused by the cytotoxicity of TGSBE on RAW264.7 macrophage cells.

### Effects of TGSBE on LPS-induced NO production in RAW264.7 cells

**Figure 1.**
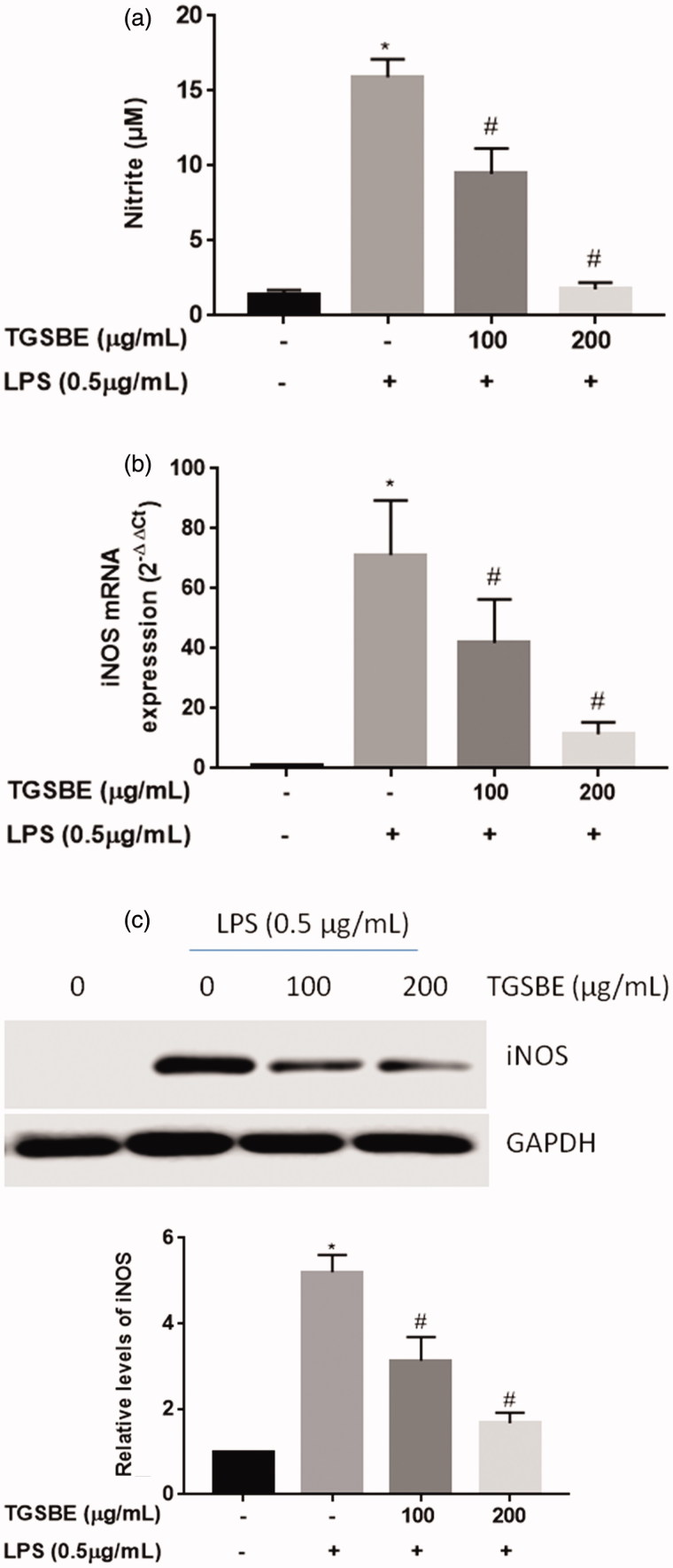
Effect of TGSBE on LPS-induced NO production and iNOS expression in RAW264.7 cells. (a) cells were treated with indicated concentrations of TGSBE for 1 h, followed by LPS (0.5 μg/mL) for 24 h. Afterwards, cell-free supernatant was collected and the amount of NO was quantified by Griess method using a standard solution of sodium nitrite prepared in the same cell-culture medium. (b) the mRNA levels of iNOS was determined by RT-PCR. (c) Whole cell lysates were subjected to SDS-PAGE and the protein levels of iNOS was determined by Immuno-blot analysis. Data represent mean ± SD from three separate experiments. *p < 0.05, significant compared to control, #*p* < 0.05, significant compared to LPS alone treated group.

Initially, to assess the anti-inflammatory effect of TGSBE, the impact of TGSBE on NO production in LPS-challenged RAW264.7 cells was analyzed. When cells were exposed to LPS for 24 h, the production of NO was dramatically increased. However, treatment with TGSBE markedly reduced NO production (Figure 1(a)). There was around 40.56% and 88.99% reduction in LPS-induced NO production at 100 and 200 μg/mL concentration of TGSBE, respectively. Further, we determined the levels of respective mRNA and protein expression in RAW264.7 cells; persistent enhanced expression of iNOS leads to increased production of NO, which eventually results in progression of inflammatory responses. As depicted in Figure 1(b,c), mRNA and protein expressions of iNOS were dramatically increased in RAW264.7 cells by LPS-stimulation, compared to the LPS absent group. However, co-treatment with TGSBE induced a lowering of the transcriptional and translational levels of iNOS. The inhibitory effects of TGSBE were dose-dependent; this result was consistent with the results of NO quantification (Figure 1(a)).

### Modulatory effects of TGSBE on pro-inflammatory cytokine production in LPS-stimulated macrophages

**Figure 2.**
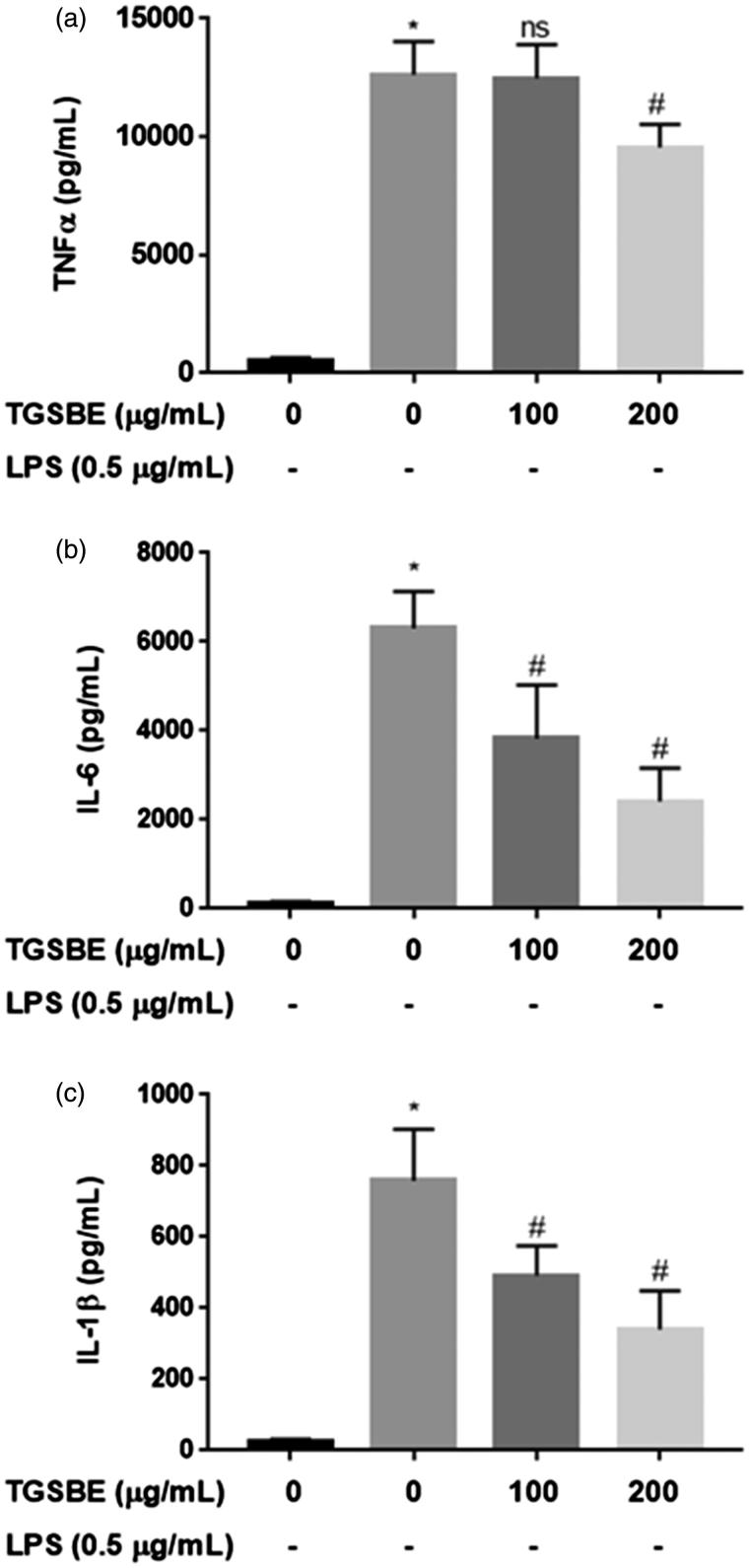
Effect of TGSBE on LPS-induced cytokine production in RAW264.7 cells. The cells were treated with indicated concentrations of TGSBE for 1 h, followed by LPS (0.5 μg/mL) for 24 h. Then, cell-free supernatant was subjected to quantify TNFα, IL-6 and IL-1β levels, using ELISA kits, according to manufacturer’s protocol. Data represent mean ± SD from three separate experiments. *p < 0.05, significant compared to control, #*p* < 0.05, significant compared to LPS alone treated group.

**Figure 3.**
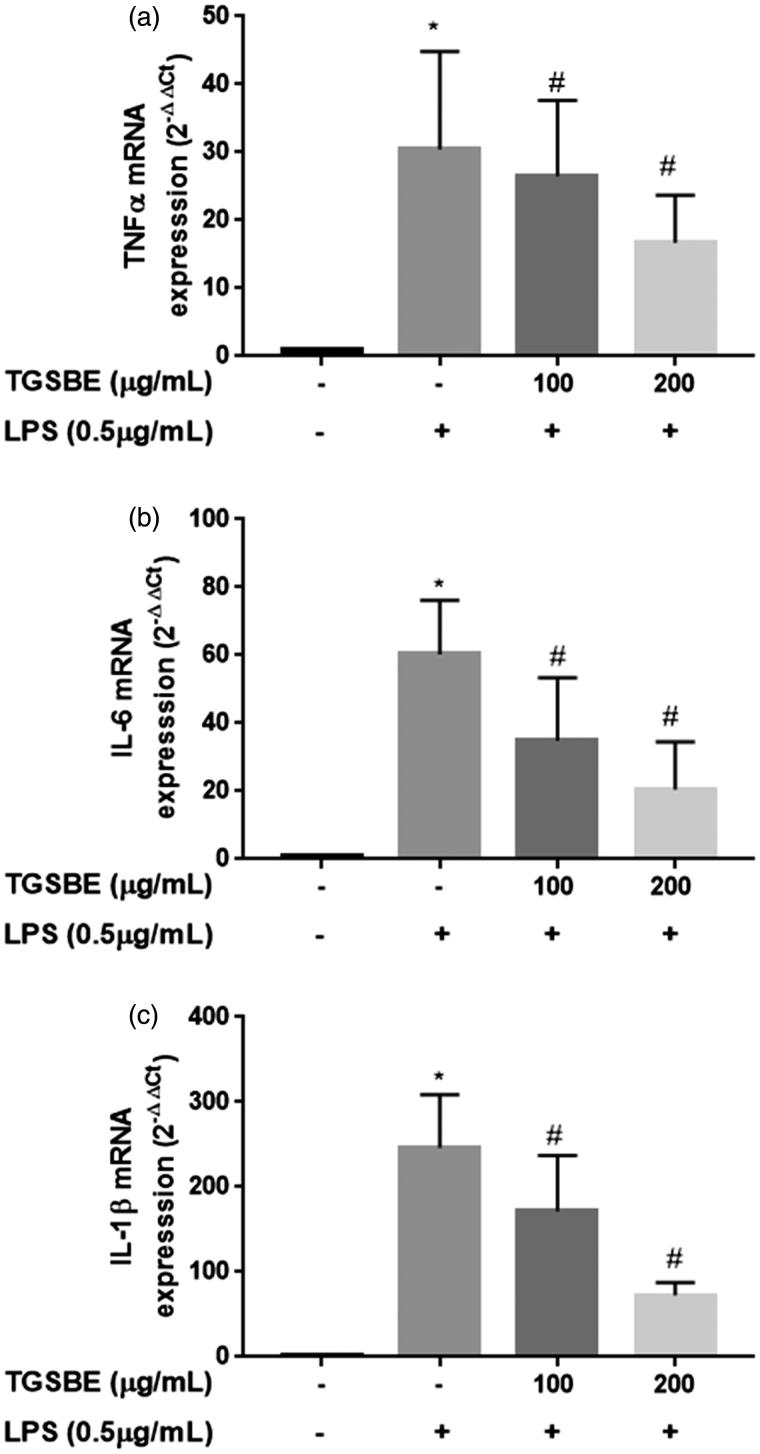
Effect of TGSBE on LPS-induced mRNA expression of TNFα, IL-6 and IL-1β in RAW264.7 cells. The cells were pre-incubated with indicated concentrations of TGSBE for 1 h, followed by LPS (0.5 μg/mL) for 12 h. Then, total RNA was extracted and mRNA expression of TNFα, IL-6 and IL-1β was analyzed by real-time RT-PCR. Data represent mean ± SD from three separate experiments. *p < 0.05, significant compared to control, #*p* < 0.05, significant compared to LPS alone treated group.

Next, the inhibitory effect of TGSBE on the production of pro-inflammatory cytokines was investigated in LPS-challenged RAW264.7 macrophages. Upon stimulation of LPS, macrophages produce hundreds of cytokines, among which TNFα, IL-6 and IL-1β are of particular interest due to their contribution to the amplification of inflammatory responses. As shown in Figure 2 the stimulation of macrophages with LPS for 24 h significantly increased the production of TNFα, IL-6 and IL-1β, by 24.2-, 47.0-, and 28.3-fold, respectively, compared to the control group, as determined by the ELISA method. However, co-treatment with TGSBE dose-dependently decreased the production of pro-inflammatory cytokines TNFα, IL-6 and IL-1β, compared to cells treated with LPS alone. The maximum inhibitory effect of TGSBE on cytokines TNFα, IL-6 and IL-1β production was 24.38, 61.70, and 55.12%, at a 200 μg/mL concentration, respectively. Further, the transcriptional levels of cytokines were analyzed by quantitative real time PCR using specific primers and SYBR green DNA binding dye in reaction mixtures. As depicted in Figure 3, LPS stimulation shows a 30.5-, 60.3-, and 246.3-fold increase in mRNA expression of TNFα, IL-6 and IL-1β, respectively, compared to the control group. Co-treatment with TGSBE induced a significant inhibitory effect on the expression of cytokines. TGSBE at a concentration of 200 μg/mL, showed 45.24, 65.84, and 70.48% reduction in mRNA expressions of TNFα, IL-6 and IL-1β, respectively.

### Effect of TGSBE on LPS-induced NF-κB activation in RAW264.7 macrophages

**Figure 4.**
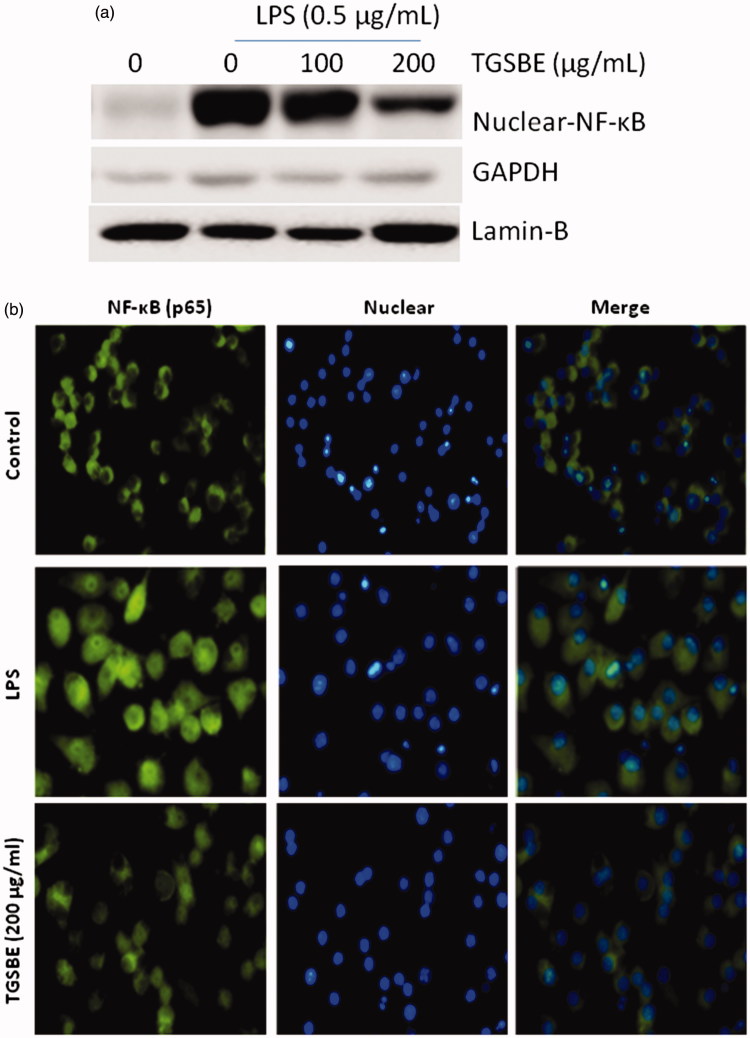
Effect of TGSBE on LPS-induced nuclear translocation of NF-κB p65 in RAW264.7 cells. (a) Cells were pretreated with indicated concentrations of TGSBE for 12 h, prior to stimulation of LPS (0.5 μg/mL) for 30 min. Nuclear fractions were collected and subjected to immunoblotting of NF-κB p65, Lamin-B and GAPDH. (b) The translocation of NF-κB p65 to the nucleus was analyzed by confocal microscopy. After treatment, cells were permeabilized and immunostained with NF-κB p65 for overnight. Followed by conjugation with FITC tagged secondary antibody and Hoechst for nucleus. Magnification, 200x. Images are representative of three separate experiments. The fluorescence of NF-κB p65 protein and nuclei is green and blue, respectively.

It has been well documented that activation and nuclear translocation of the transcription factor, NF-κB, plays a critical role in the expression of various pro-inflammatory mediators. LPS is known to be responsible for activation and nuclear translocation of NF-κB p65 in many cell types, including macrophages. Hence, to assess the effect of TGSBE on LPS-induced activation of NF-κB, RAW264.7 macrophage cells were pretreated with TGSBE for 12 h and then stimulated with LPS for 30 min. Afterwards, nuclear fractions were collected and immunoblotted for the NF-κB p65 subunit using a specific antibody. As shown in Figure 4(a,b), it is clear that LPS stimulation led to an increase of NF-κB p65 levels in the nucleus while treatment with TGSBE showed significantly decreased levels of this protein. The absence of the housekeeping protein, GAPDH, confirms the lack of any cytosolic fraction in the nuclear extracts (Figure 4(a)).

### TGSBE inhibits LPS-induced MAPKs activation in RAW264.7 macrophages

**Figure 5.**
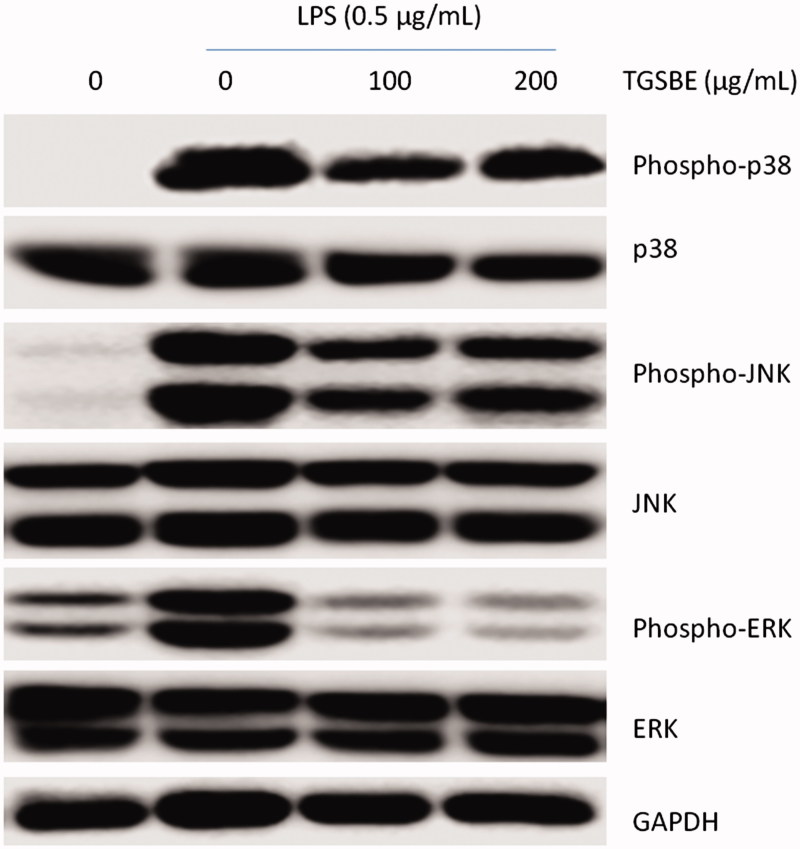
Effect of TGSBE on LPS-induced activation of MAPKs in RAW264.7 cells. Cells were pre-incubated with TGSBE for 12 h, followed by LPS stimulation for 30 min. Then, whole cell lystaes were prepared and used to analyze the levels of phosphorylated and non-phosphorylated p38, JNK or ERK1/2 by immune-blot assay. Blots are representative of three independent experiments.

In addition to the NF-κB signaling pathway, LPS also activates MAPKs to regulate the synthesis and secretion of inflammatory mediators in macrophages as well as many other cell types. Therefore, we investigated the influence of TGSBE on LPS induced activation of MAPKs such as p38, JNK and ERK. Macrophages were stimulated with LPS for 30 min, after pretreatment with or without TGSBE for 12 h. Then, whole cell lysates were prepared and immunoblotted using specific antibodies. As depicted in Figure 5, phosphorylation, and thereby activation, of p38, JNK and ERK were increased by LPS stimulation. TGSBE significantly attenuated the LPS-induced activation of all three MAPKs in macrophages.

### TGSBE inhibits LPS-induced ROS production in RAW264.7 macrophages

**Figure 6.**
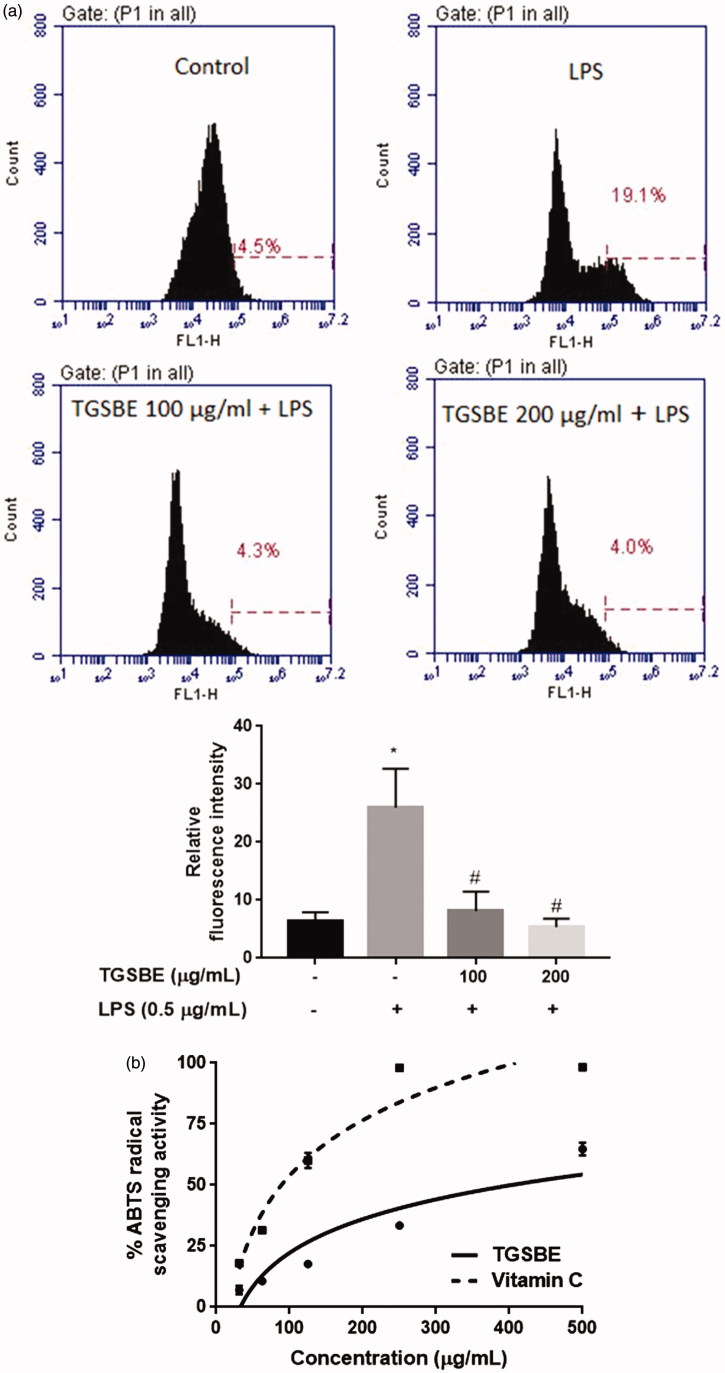
(a) Effect of TGSBE on LPS-induced production of ROS in RAW264.7 cells. Following treatment with TGSBE and LPS, cells were incubated with DCF-DA for 30 min. Afterwards, cells were collected and fluorescent intensity which is proportional to ROS concentration, was determined by flow-cytometry. Images are representative of three separate experiments. The relative level of ROS production was depicted in graph. Data represent mean ± SD values of three separate experiments. *p < 0.05, significant compared to control, #*p* < 0.05, significant compared to LPS alone treated group. (b) the antioxidant potential of TGSBE was determined by ABTS radical scavenging assay. The representative graph depicts the percentage ABTS radical scavenging potential of TGSBE and a reference standard ascorbic acid. Data represent mean ± SD values of three separate experiments.

ROS are known to be critical for the LPS-stimulated activation of MAPKs and NF-κB signaling pathways as well as induction of mitochondrial membrane depolarization. To investigate the effect of TGSBE on LPS-induced ROS production, we determined the levels of ROS in RAW264.7 murine macrophage cells using the fluorescent probe DCF-DA. The cells were first treated with TGSBE for 1 h, then, selectively exposed to LPS for 12 h. Afterwards, cells were incubated with the fluorescent probe, DCF-DA at a final concentration of 10 μM for 30 min. The amount of DCF-DA oxidized by intracellular ROS was determined by assaying the fluorescence intensity of oxidized DCF-DA which is proportional to the concentration of ROS, using flow cytometry. Stimulation of macrophages with LPS showed a marked increased level of ROS compared to control group. TGSBE treatment significantly reduced the ROS concentration in LPS-induced macrophages (Figure 6(a)). This activity of TGSBE may be due its antioxidant property evidenced from the ABTS radical scavenging assay (Figure 6(b)).

### TGSBE inhibits LPS-induced mitochondrial membrane depolarization in RAW264.7 macrophages

**Figure 7.**
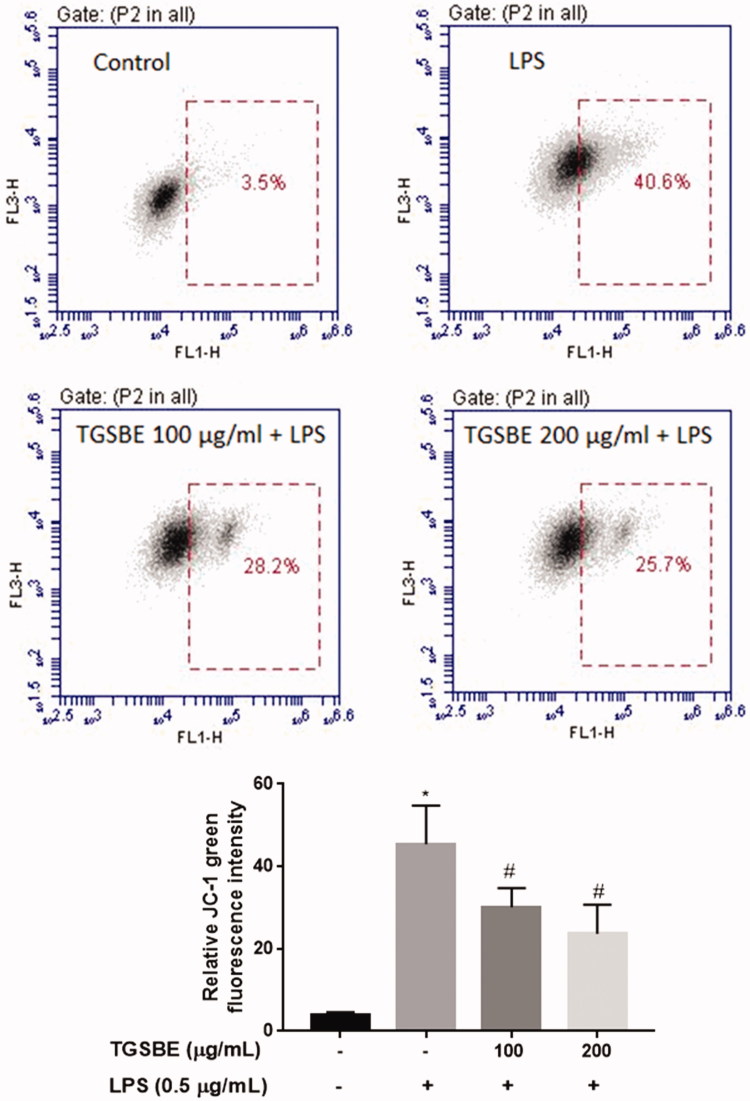
Effect of TGSBE on LPS-induced loss of mitochondrial membrane potential in RAW264.7 cells. Cells were pretreated or not with TGSBE for 1 h and exposed or not to LPS for 12 h. Cells were harvested and incubated with JC-1 for 15 min. After removing the excess JC-1, cells were analyzed using a flow-cytometer. Images are representative of three separate experiments. The relative level of loss of mitochondrial membrane potential was depicted in graph. Data represent mean ± SD values of three separate experiments. *p < 0.05, significant compared to control, #*p* < 0.05, significant compared to LPS alone treated group.

Upon stimulation of macrophages with LPS, the increased ROS and MAPKs activation damages the mitochondrial membrane integrity. To examine the impact of TGSBE on LPS-induced loss of mitochondrial membrane integrity, we stained the cells with the mitochondrial specific dye JC-1 and analyzed the cells using flow cytometry. JC-1 fluoresces, red if the membrane potential is normal and fluoresces green when the membrane potential has been disturbed. Figure 7 demonstrates the loss of mitochondrial membrane potential in LPS-stimulated macrophages. This was significantly attenuated by TGSBE at both minimum and maximum concentrations.

## Discussion

Nitric oxide (NO) is an important intracellular signaling molecule produced by various cell types including immune cells. Under physiological levels, NO plays an important role in various processes such as the host defensive response against pathogens, neuronal communication and vasodilation (Bogdan et al. 2000). However, the increased production of NO in some abnormal situations has been reported to induce systemic tissue damage (Abramson et al. 2001). It is well-known that many acute and chronic inflammatory diseases are associated with increased production of NO in inflammatory cells like macrophages (Chin et al. 2008). Therefore, inhibition of NO production in macrophages is one of the possible ways to control the severity of tissue damage in inflammatory diseases. There are three different isoforms of NO synthase (NOS) enzymes that exist in mammalian cells: endothelial NO synthase (eNOS), neural NO synthase (nNOS) and inducible NO synthase (iNOS). The major isoform of NO synthase expresses in macrophages is iNOS (Panaro et al. 2003). LPS, a component of the cell wall of gram negative bacteria is a powerful activator of iNOS expression in macrophages (Aldridge et al. 2008). Hence, we analyzed the effect of TGSBE on LPS-induced expression of iNOS in RAW264.7 murine macrophage cells. TGSBE treatment showed significant, decreased levels of LPS-induced iNOS expression in murine macrophage cells, which was consistent with the reduced levels of NO, observed in our study.

LPS binds to toll-like receptors (TLR) on macrophages and triggers the activation of various inflammatory pathways including NF-κB. The transcription factor, NF-κB, exists in various isoforms either as homo- or heterodimers. The major isoform of NF-κB is a heterodimer of p65 and p50. NF-κB is generally located in the cytoplasm with its inhibitory protein IκBα. Upon stimulation by LPS, NF-κB is released from its inhibitory protein, IκBα, due to phosphorylation, ubiquitination and subsequent degradation of IκBα by proteasome. The relaeased, IκBα free, NF-κB is, then, translocated to the nucleus where it induces the expression of various pro-inflammatory genes such as iNOS, COX-2, TNFα, IL-6 and IL-1β (Fujihara et al. 2003; Hong Byun et al. 2010; Wang et al. 2016). In the present study, therefore, immunoblotting was conducted to analyze the effect of TGSBE on the nuclear translocation of NF-κB in LPS-stimulated RAW264.7 macrophage cells. We found that LPS alone markedly increased the nuclear levels of NF-κB whereas TGSBE significantly reduced the LPS-induced, increased levels of NF-κB in the nucleus.

In addition to NF-κB signaling, activation of MAPKs pathways by LPS also stimulates an inflammatory response in macrophages by increasing the synthesis and secretion of inflammatory mediators (Fang et al. 2004). MAPKs are intracellular serine/threonine protein kinases that exist as three different kinases, namely, p38 mitogen-activated protein kinase (p38 MAPK), c-Jun NH_2_-terminal kinase (JNK) and extracellular signal regulated kinase 1/2 (ERK1/2). Studies have shown that inhibition of all three MAPKs in LPS-stimulated macrophages leads to decrease the production of pro-inflammatory mediators (Yun et al. 2008; Ci et al. 2010; Guo et al. 2012). Furthermore, inhibition of p38 MAPK alone inhibits the activity of NF-κB by various mechanisms (Carter et al. 1999). In the present study, the decreased NF-κB activity and production of inflammatory mediators by TGSBE in LPS-stimulated macrophages might be partly due to the inhibitory effects of TGSBE on MAPKs.

The production of ROS molecules, especially superoxide anion radical and hydrogen peroxide is indispensable for the life of aerobic organisms. Superoxide anion radicals are known to regulate various physiological functions such as cell growth, differentiation, aging, senescence and apoptosis (Chiste et al. 2015). Hydrogen peroxide plays an important role in the degradation of some proteins, acts as a messenger in cell signaling and also contributes to apoptotic cell death (Góth 2006). However, these ROS molecules cause injury to tissues at higher concentrations as they oxidize and impair the functions of important cellular components as well as activate stress signaling pathways (Evans et al. 2003; Zuo et al. 2015). During inflammatory disease conditions, the enhanced ROS production from inflammatory cells, like macrophages, in response to various stimuli increases the severity of tissue damage. LPS increases the production of ROS in macrophages that further activates the inflammatory signaling pathways, NF-κB and MAPKs, as well as damages the mitochondrial membrane integrity (Hsu & Wen 2002; Bognar et al. 2013). Our study demonstrates that TGSBE treatment significantly decreased the concentration of ROS in LPS-induced RAW264.7 macrophage cells. The reduced ROS levels in TGSBE treated cells might be partly responsible for the reduced activity of NF-κB and MAPKs and the improved mitochondrial membrane potential.

Plant-derived secondary metabolites like phenolic compounds have been reported to have the potential of inhibiting inflammatory reactions via suppression of NFκB and MAPKs pathways (Kazłowska et al. 2010; Costa et al. 2012). Hence, we speculate that the presence of phenolic compounds in TGSBE might be responsible for the anti-inflammatory activity.

## Conclusion

The results of this study showed that TGSBE significantly inhibited the production of NO, TNFα, IL-6 and IL-1β as well as inhibited the respective gene expression of these proteins in LPS-stimulated RAW264.7 macrophages. In addition, TGSBE improved the mitochondrial membrane potential in LPS-induced cells. These effects may be as a result of the inhibitory action of TGSBE on LPS-induced production of ROS, and the activation of NF-κB and MAPK signaling pathways. Collectively, we for the first time reported that *Ternstroemia gymnanthera* stem bark extract possesses significant anti-inflammatory effects in LPS-stimulated RAW264.7 murine macrophage cells.
